# A novel endophytic fungus strain of *Cladosporium*: its identification, genomic analysis, and effects on plant growth

**DOI:** 10.3389/fmicb.2023.1287582

**Published:** 2023-11-23

**Authors:** Nan Yang, Wenbin Zhang, Dan Wang, Dingding Cao, Yanyu Cao, Weihong He, Ziting Lin, Xiaofeng Chen, Guiping Ye, Zhiming Chen, Jianjun Chen, Xiangying Wei

**Affiliations:** ^1^Fujian Key Laboratory on Conservation and Sustainable Utilization of Marine Biodiversity, Fuzhou Institute of Oceanography, College of Geography and Oceanography, Minjiang University, Fuzhou, Fujian Province, China; ^2^Institute of Cytology and Genetics, Hengyang Medical School, University of South China, Hengyang, Hunan, China; ^3^Mid-Florida Research and Education Center, Department of Environmental Horticulture, Institute of Food and Agricultural Sciences, University of Florida, Apopka, FL, United States

**Keywords:** *Cladosporium*, genome sequencing, IAA, plant growth-promoting fungi (PGPF), BF-F, PGP gene clusters

## Abstract

**Introduction:**

Endophytic microorganisms are bacteria or fungi that inhabit plant internal tissues contributing to various biological processes of plants. Some endophytic microbes can promote plant growth, which are known as plant growth-promoting endophytes (PGPEs). There has been an increasing interest in isolation and identification of PGPEs for sustainable production of crops. This study was undertaken to isolate PGPEs from roots of a halophytic species *Sesuvium portulacastrum* L. and elucidate potential mechanisms underlying the plant growth promoting effect.

**Methods:**

Surface-disinfected seeds of *S. portulacastrum* were germinated on an *in vitro* culture medium, and roots of some germinated seedlings were contaminated by bacteria and fungi. From the contamination, an endophytic fungus called BF-F (a fungal strain isolated from bacterial and fungal contamination) was isolated and identified. The genome of BF-F strain was sequenced, its genome structure and function were analyzed using various bioinformatics software. Additionally, the effect of BF-F on plant growth promotion were investigated by gene cluster analyses.

**Results:**

Based on the sequence homology (99%) and phylogenetic analysis, BF-F is likely a new *Cladosporium angulosum* strain or possibly a new *Cladosporium* species that is most homologous to *C. angulosum*. The BF-F significantly promoted the growth of dicot *S. portulacastrum* and Arabidopsis as well as monocot rice. Whole genome analysis revealed that the BF-F genome has 29,444,740 bp in size with 6,426 annotated genes, including gene clusters associated with the tryptophan synthesis and metabolism pathway, sterol synthesis pathway, and nitrogen metabolism pathway. BF-F produced indole-3-acetic acid (IAA) and also induced the expression of plant N uptake related genes.

**Discussion:**

Our results suggest that BF-F is a novel strain of *Cladosporium* and has potential to be a microbial fertilizer for sustainable production of crop plants. The resulting genomic information will facilitate further investigation of its genetic evolution and its function, particularly mechanisms underlying plant growth promotion.

## 1 Introduction

Many microorganisms, particularly fungi and bacteria that can colonize intercellular spaces, epidermis, endodermis, and vascular bundles of host plant tissues or inside the cells for their entire or partial life cycle without causing damage to the host plant. These microbes are defined as endophytes (Saikkonen et al., 2003; Perotto et al., 2012). Endophytes are considered as a symbiotic association with host plants. Plants from nearly all the genera of the Kingdom Plantae harbor fungal endophytes within their internal tissues. Plants with endophytic fungi have been shown to be more resistant to pathogens, better utilize resources, and exhibit increased productivity (Baron and Rigobelo, 2021). Endophytic fungi can protect plants from pathogen infections by either direct inhibition through competition, antibiosis or mycoparasitism or indirect inhibition through enhancing resistance (Latz et al., 2018; Silva et al., 2018; Ebrahimi et al., 2022; Wei et al., 2022). Many protective secondary metabolites have been identified from endophytic fungi, such as flavonoids, terpenoids, alkaloids, phenols, volatile organic compounds (VOC). Some bioactive compounds identified from endophytic fungi can be used for drug discovery and agricultural applications for controlling pathogens and insect pest (Zhang et al., 2006; Meenavalli et al., 2011; Gouda et al., 2016; Toghueo et al., 2017; Strobel, 2018). In addition, some endophytic fungi inside host plants are capable of degrading a portion of plant lignin and cellulose which helps the host plant’s defense against invasive pathogens (Marques et al., 2018; Li et al., 2021) or secrete extracellular chitinase to decompose chitin and damage the cell wall structure of phytopathogenic fungi (Hartl et al., 2012; Kumar et al., 2018).

Endophytes also contribute to multiple bioprocesses of plant growth and development, including nitrogen fixation, phosphate solubilization, and phytohormones production (Santoyo et al., 2016; Priyadharsini and Muthukumar, 2017; Zhang et al., 2018). Many endophytic fungi possess phosphate solubilizing activity, such as *Pestalotiopsis*, *Trichoderma, Penicillium,* and *Aspergillus* species (Zhang et al., 2016; Hossain et al., 2017; Adhikari and Pandey, 2019; Erman et al., 2022). The solubilization of inorganic phosphate by endophytes has been shown to contribute significantly to the bioavailability of phosphorus (Adhikari and Pandey, 2019). Phytohormones are important plant signaling compounds regulating plant growth and development as well as adaptation to stressful conditions. Many endophytic microbes can synthesize indole-3-acetic acid (IAA), gibberellins (GAs), or some cytokinin compounds (Hamayun et al., 2009; Aly et al., 2010; Sasirekha et al., 2012; You et al., 2013; Khan et al., 2014, 2015; Wei et al., 2020). In the plant growth-promoting endophytic fungi group, some can secrete GAs or IAA to regulate host plant growth and developmental (Hamayun et al., 2009; Shah et al., 2018). Since endophytic fungi not only act as biocontrol agents but as biostimulants and biofertilizers, a considerable effort has been placed on the isolation of endophytic fungi from different plants and identification of their role in improving plant growth and/or adaptation to abiotic and biotic stresses.

*Cladosporium* fungi belong to the order Capnodiales in the class Dothideomycete and a member of the dematiaceous hyphomycetes (Dugan et al., 2004; Bensch et al., 2018). *Cladosporium* is commonly found in plant, fungal, and other organic debris, can be isolated from soil, air, food, paint, textiles, and diverse types of organic materials (Farr et al., 1989; Flannigan, 2001; Mullins, 2001; Sandoval-Denis et al., 2015). In plants, *Cladosporium* species are commonly known as endophytes (El-Morsy, 2000; Hamayun et al., 2009; Venkateswarulu et al., 2018) and phylloplane fungi (Levetin and Dorseys, 2006). However, the effects of *Cladosporium* on plants are diverse. Some *Cladosporium* species are pathogens that cause plant disease (Heuchert et al., 2005; Watson and Napier, 2009; Kryczyński and Weber, 2011; Ogórek et al., 2012), and some other species are beneficial to plants by promoting plant growth (Hamayun et al., 2009; Paul and Park, 2013; Sandoval-Denis et al., 2015) or enhancing plant tolerance to biotic and abiotic stresses (Chaibub et al., 2016; Torres et al., 2017).

The beneficial effects of *Cladosporium* on plants include the release of some secondary metabolites for improving plant growth (Hamayun et al., 2009; Paul and Park, 2013). Thus, the analysis of the metabolic pathways of *Cladosporium* species will help define species functions within *Cladosporium*. Advances in sequencing technology have accelerated fungal genomic studies, and new third-generation sequencing techniques allow researchers to describe the molecular diversity of more closely related strains (Xu et al., 2021). Clusters of genes for secondary metabolic pathways are an emerging theme in plant biology and microbiology, which provides some provocative insights into genome plasticity and evolution (Osbourn, 2010). The potential functions of *Cladosporium* species in plants can be quickly predicted based on the gene clusters of secondary metabolic pathways obtained from the genome.

Halophytic *Sesuvium portulacastrum* L. is a pioneer plant species used for sand-dune fixation, desalination, and phytoremediation along coastal regions of Asia, Arabian Peninsula, and South China due to its tolerance to salinity. It is also used as a vegetable, ornamental plant, and fodder for domestic animals (Lokhande et al., 2013). The extracts of *S. portulacastrum* are widely used as essential oils and folk medicine (Lokhande et al., 2013; Patel et al., 2019). There is a speculation that its salt tolerance and medicinal properties may be related to endophytic and rhizosphere microorganisms. A halotolerant PGPR isolated from *S. portulacastrum* was found to improve salt tolerance of *Vigna mungo* L. (John et al., 2023). Endophytic and rhizosphere fungi isolated from *S. portulacastrum* increased cucumber plants tolerance to damping off caused by *Pythium aphanidermatum* (Karunasinghe et al., 2020). Therefore, application of endogenous microorganisms isolated from halophytes, such as *S. portulacastrum* could be a strategy to improve crop plants to tolerate salinity and increase crop productivity.

We recently isolated an endophytic fungus from roots of *in vitro* cultured *S. portulacastrum* seedlings, which was able to substantially enhance plant growth. The objectives of this study were to identify and characterize this isolate, evaluate its effects on plant growth, perform genomic sequencing, analyze hormones in mycelium, and determine likely mechanisms underlying the plant growth promoting effects.

## 2 Materials and methods

### 2.1 Isolation of an endophytic fungus from *Sesuvium portulacastrum*

Seeds of *S. portulacastrum* were disinfected with 75% (v/v) ethanol for 1 min, and then in 3% NaClO (v/v) for 6 min. After rinse with sterile distilled water three times, the seeds were placed on Murashige and Skoog (MS) medium (Murashige and Skoog, 1962) supplemented with 3% (w/v) sucrose and 0.6% (w/v) agar at 25°C for germination. The MS has been the most widely used plant tissue culture medium since 1962.

After germination, we found that roots of few seedlings appeared to be contaminated with fungi and bacteria, and the contaminated seedlings were taller and stronger than those uncontaminated. Using the methods described by Khan et al. (2015) and Wei et al. (2016), a fungus was isolated. Briefly, roots of contaminated seedlings were disinfected as mentioned above and cut into about 0.5 cm pieces and placed on a water agar (WA) medium and incubated in the dark at 25°C until fungal growth started. The fungus was then cultured on malt extract agar (MEA) solid medium (1 g/L tryptone, 20 g/L malt extract, 20 g/L glucose, and 20.0 g/L agar) for morphological identification. The colony characteristics of this fungus were determined after growing on solid potato dextrose agar (PDA) medium (6.0 g/L potato powder +20.0 g/L glucose +20.0 g/L agar; pH = 5.6–5.8) for 7 days in the dark at 25°C (described below). This isolate was stored in the Chinese Center for Microbial Species Preservation and Management (CGMCC NO: 23053) and referred to as BF-F (a fungus isolated from bacterial and fungal contaminated culture) in the library.

### 2.2 Morphological identification of *Cladosporium* BF-F

Morphological characteristics of BF-F, including colony diameter, color, pigments, and reverse side of colony color, were examined after culturing on PDA for 7 days in the dark at 25°C. Conidia and conidiophores were induced using 20% potato dextrose broth, a small volume (about 50 μL) of 20% potato dextrose broth was dropped into each of two holes of a glass slide placed in a 9 cm plastic Petri dish, hyphae of isolate were added, and then incubated in the dark at 25°C for 7–21 days. Conidia were observed under a light microscope. Hypha was able to separate and branch to 2.0–5.5 μm in width. The fungus was putatively identified with taxonomic keys in Barron (1962) and Domsch et al. (1980).

### 2.3 Molecular identification of *Cladosporium* BF-F

The isolate of BF-F was cultured in a liquid PDB medium with shaking (120 r/min) at 25°C for 14 days. The culture was collected in sterile 50 mL centrifuge tube by centrifuging at 10,000 × g for 10 min and then frozen in liquid nitrogen. After grinding the fungi with a mortar and pestle. Genomic DNA was extracted using the SDS method (Lim et al., 2016). The isolated DNA was confirmed by agarose gel electrophoresis and quantified by Qubit^®^ 2.0 Fluorometer. The internal transcribed spacer (ITS) region was amplified using the ITS1 (5’ TCCGTAGGTGAACCTGCGG 3′) and ITS4 (5’ TCCTCCGCTTATTGATATGC 3′) primers. The amplifications were performed in a 50 μL reaction volume containing 50 ng of genomic DNA, 50 pmol of each primer, 100 μM of dNTP, 1 U of Taq polymerase, and 5 μL of 10 × PCR buffer. The tubes were incubated at 95°C for 2 min and then subjected to 35 cycles as follows: 94°C for 30 s, 60°C for 30 s, and 72°C for 45 s; a final incubation was carried out for another 5 min at 72°C. The PCR products were cloned with the PMD19-T Easy Vector System and analyzed by an ABI 3730XI automatic DNA Sequencer. The molecular identification was carried out using the ITS region and *EF-1α* sequence (Supplementary Table S1). The isolate was subjected to DNA sequencing and ITS-based phylogenetic analysis as previously described with slight modification (Yew et al., 2014). The BLAST search was conducted for the maximum percentage of sequence homology and query coverage as well as the lowest E values. The rDNA ITS sequences of species in *Cladosporium* were searched for in the UNITE database (Nilsson et al., 2019), and the search parameters used were as follows: “Threshold” was “1.5″; “include” was “All SH-s.” The complete ITS1-5.8S-ITS4 and *EF-1α* sequences for 12 *Cladosporium* species were obtained from GenBank for phylogenetic tree construction with BF-F. Neighbor-joining tree analysis was performed using Molecular Evolutionary Genetics Analysis (MEGA) software with 1,000 bootstrap replications.

### 2.4 Effects of *Cladosporium* BF-F on plant growth

The effect of BF-F on plant growth was determine on *S. portulacastrum, Arabidopsis thaliana*, and rice (Tp309), respectively. Seeds of the three plants were disinfected in 75% (v/v) ethanol for 1 min and then in 3% NaClO (v/v) for 6 min. After rinse with sterile distilled water three times, the *S. portulacastrum* seeds were placed on MS medium at 25°C for germination. The germination took place in a growth room under white-fluorescent light with 16 h light and 8 h dark for 14–16 days. Seedlings were inoculated with a diameter 0.5 cm BF-F fungus colony as described by Wei et al. (2016). Plant height, root number, and leaf number of the *S. portulacastrum* seedlings were recorded after 1–4 weeks of co-cultivation. The same procedure was used for germination of *A. thaliana* and rice seeds. BF-F effects on *Arabidopsis* and rice seedling growths were evaluated after 14 and 28 days of inoculation, respectively.

### 2.5 Genome sequencing and assembly

The genome of BF-F strain was sequenced and assembled by Beijing Novogene Technology Co., Ltd. (Beijing, China) using the Illumina Novaseq 6,000 and PacBio Sequel I high-throughput sequencing platform (Beijing Nuohe Zhiyuan Technology Co., Ltd., Beijing, China). The obtained raw data were filtered to obtain high-quality DNA sequence data and then processed in accordance with the platform gene assembly process. The third-generation sequencing data generated by PacBio Sequel I Sequencing, SMRT Link v5.0.1 software was used to assemble the reads to obtain preliminary assembly results that reflected the basic situation of the sample genome, and the WTDBG software assembly results were selected for subsequent processing. PacBio Sequel I Arrow software (Chaisson and Tesler, 2012) was then used to perform self-single base correction. With the use of the variant Caller module of SMRT Link software, the arrow algorithm was used to correct and count the variant sites in the preliminary assembly results, and Pilon software (Walker et al., 2014) was used to correct the sequence assembled by the third-generation sequencing and to finally obtain the corrected genome. The sequence was submitted to the NCBI database under the accession number JAMRMS000000000.

### 2.6 Functional genome annotation

The Gene Ontology Resource was used for Gene Ontology (GO),^1^ analysis (Ashburner et al., 2000). The Kyoto Encyclopedia of Genes and Genomes (KEGG) database^2^ was employed for the construction of metabolic pathways (Kanehisa et al., 2012). The NCBI Clusters of Orthologous Groups (COG) server^3^ was used to assign the obtained COG annotations for Eukaryotic Orthologous Groups (KOG) to functional categories (Koonin et al., 2004). Genes involved in plant growth promotion were excavated from the predicted annotated genes of the Cladosporium sp. BF-F genome.

### 2.7 Analysis of tryptophan and tryptophan metabolites in mycelium of BF-F

Tryptophan (Trp) and tryptophan metabolites were analyzed using mass spectrometry based on the method described by Krause et al. (2015). A diameter 0.5 cm BF-F colony was cultured in the flask containing potato dextrose broth medium on a rotating shaker at 150 rpm, 25°C in total darkness for 5 days. Mycelium was collected by filtration through Whatman filter paper No. 1 and frozen in liquid N (Wei et al., 2020). For analysis of tryptophan and tryptophan metabolites, 1 g mycelium was extracted by acetonitrile or methanol and diluted by diethyl ether to different concentration gradients. Tryptophan and tryptophan metabolites including indole-3-acetic acid, indole-3-butyric acid were analyzed using the Thermo Scientific™ Q Exactive™ HF hybrid quadrupole-Orbitrap mass spectrometer by Sci-Tech Inovation, Inc. (Qindao, China). CSH C18 RP column (150 × 2.1 mm, particle size of 1.7 μm) was used, and the analysis conditions were set as follows: cone/desolvation gas flow at 150/1000 L h^−1^; capillary voltage at 2.1 kV for ESI(+) and 1.5 kV for ESI(−); source/desolvation temperature at 125°C/600°C; collision energy at 12 to 30 eV; cone voltage at 10 to 40 V; and collision argon gas flow at 0.21 mL min^−1^.

### 2.8 Qrt-PCR analysis of genes related to auxin response and N absorption

Expression of key genes related to auxin biosynthesis and N uptake were investigated in *A. thaliana* seedlings with and without BF-F inoculated. Seedlings of *A. thaliana* were harvested after 2 weeks inoculation, and total RNAs were extracted using E.Z.N.A Plant RNA Kit (Omega Bio-tek, Norcross, GA, United States). The first stand cDNA was synthesized using the PrimeScript first cDNA Synthesis Kit (Takara, Dalian, China). qRT-PCR was used to analyze the expression level of auxin response genes, including *PRE1, IAA19, SAUR-AC1, GH3.3, ARF9,* and *PIN7* as well as nitrate transporter genes *NRT1.1, NRT1.7,* and *NRT2.7*. *PP2A* was used as an internal control. For a given gene, the relative expression level was performed as mean ± SD with three replicates. All primers used are listed in Supplementary Table S2.

## 3 Results

### 3.1 Morphological characteristics of BF-F strain

**Figure 1:**
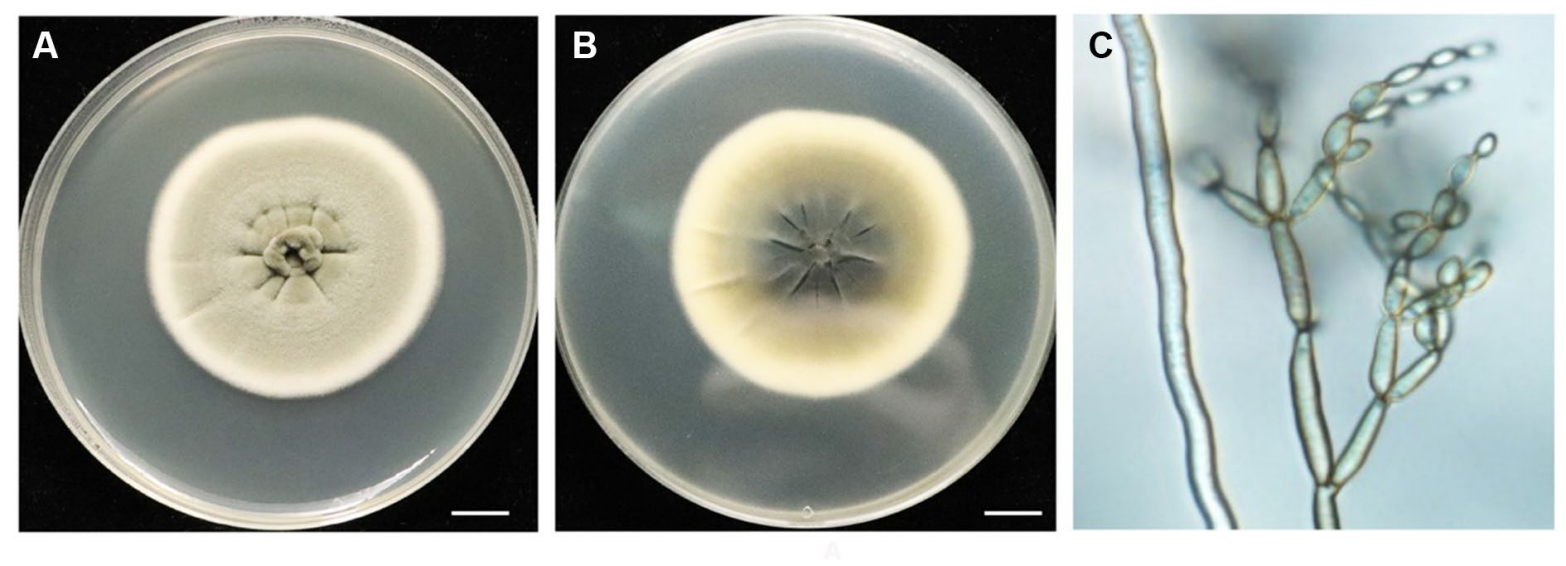
Morphological characteristics of endophytic fungi BF-F. **(A)** and **(B)** Colonial morphology of BF-F grown on PDA for 7 days at 25°C. A, front; B, back. **(C)** Light micrograph showing conidia morphology of BF-F grown on PDA for 7 days at 25°C.

The morphology of the BF-F colony exhibited grayish-green, radial wrinkles, which was velvety in texture (Figure 1A), indigo on the back, and produced no soluble pigment (Figure 1B) when cultured on MEA medium in the dark at 25°C for 7 days. The hyphae of BF-F were loose separation, concave performance, and dark brown in color (Figure 1C). Conidia were subglobose to cylindrical with light brown color under the microscope when cultured on PDA medium (Figure 1C). The color became darker with aging; conidial peduncles occurred in aerial hyphae. Their stems were divided, upright or slightly curved. Conidia were oval, 2.5–5.5 × 2–4 μm in size. Branch conidia occasionally formed, long column or oval with a size of 6–12 μm in length and 2.5–4.5 μm in width.

### 3.2 Molecular characterization

**Figure 2:**
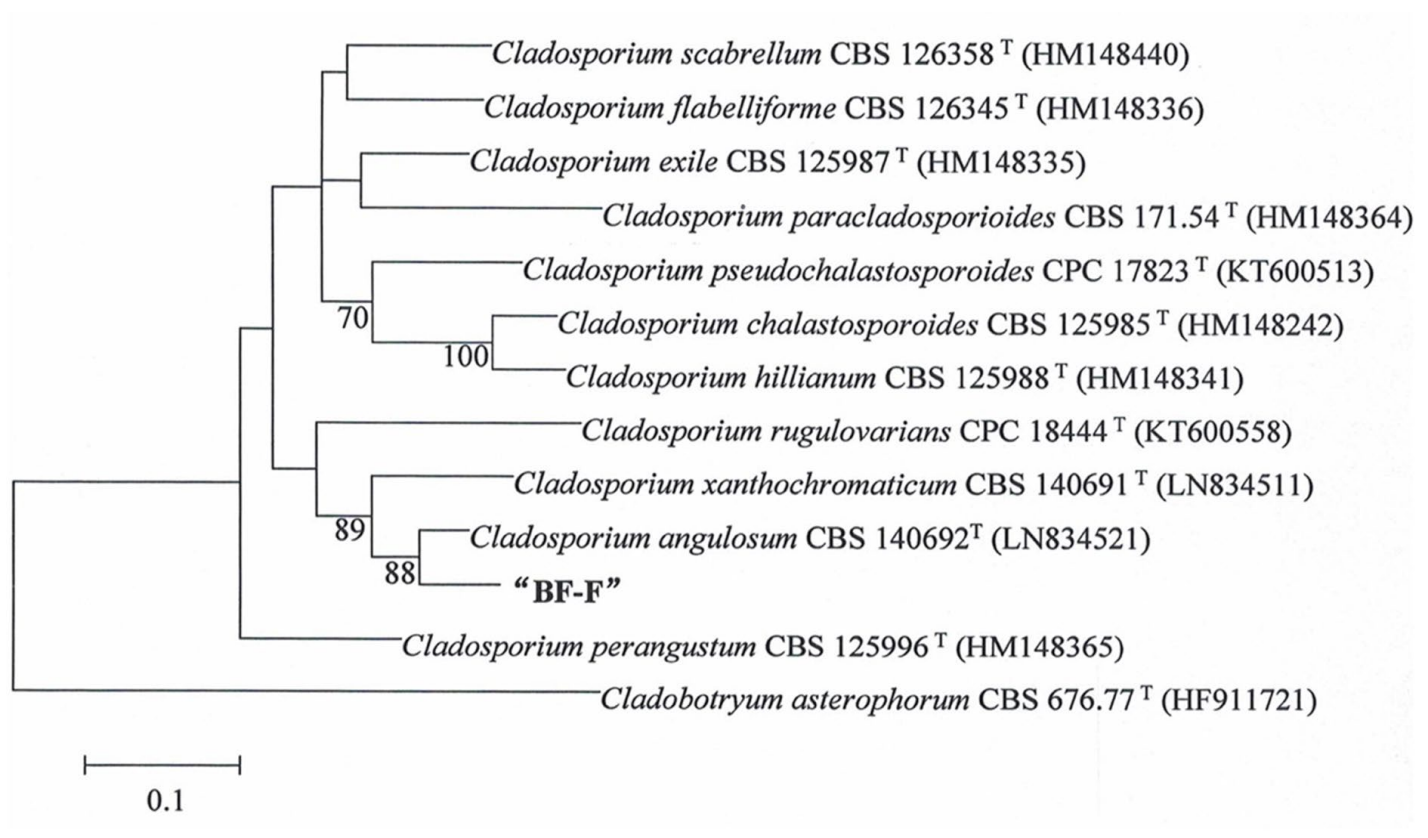
Phylogenetic tree analysis of BF-F based on *EF-1α* sequences. Tree topology of BF-F strain based on *EF-1α* sequences using the Neighbour-Joining (N-J) method. The 12 reference sequences were obtained from GenBank. Bootstrap values (calculated from1000 repetitions) ≥ 70% are shown at their relevant nodes and the superscript “T” represented the model strains.

Phylogenetic analysis of BF-F based on the ITS rDNA and *EF-1α* sequences is presented in Supplementary Table S1. The *EF-1α* and ITS rDNA sequences of BF-F were compared to the available sequences obtained by BLAST from GenBank database. Neighbor-joining trees were constructed using 13 taxa (12 references and one clone) with 1,000 bootstrap replications using MEGA software, respectively (Figure 2 and Supplementary Figure S1). The selection of these strains was based on the BLAST search as they showed the maximum percentage of sequence homology and query coverage as well as the lowest E values. In the dendrogram of the ITS rDNA sequence, BF-F fell into *Cladosporium* sp. group with strong support (Supplementary Figure S1). However, BF-F formed a monoclade with *C. angulosum* CBS140692 (type strain), which had 88% bootstrap support in the phylogenetic tree of the *EF-1α* sequence (Figure 2). Based on the sequence homology (99%) and phylogenetic analysis, BF-F may be delimited as a new strain of *C. angulosum* or a *Cladosporium* sp. homologous to *C. angulosum*.

### 3.3 Effects of *Cladosporium* BF-F on plant growth

**Figure 3:**
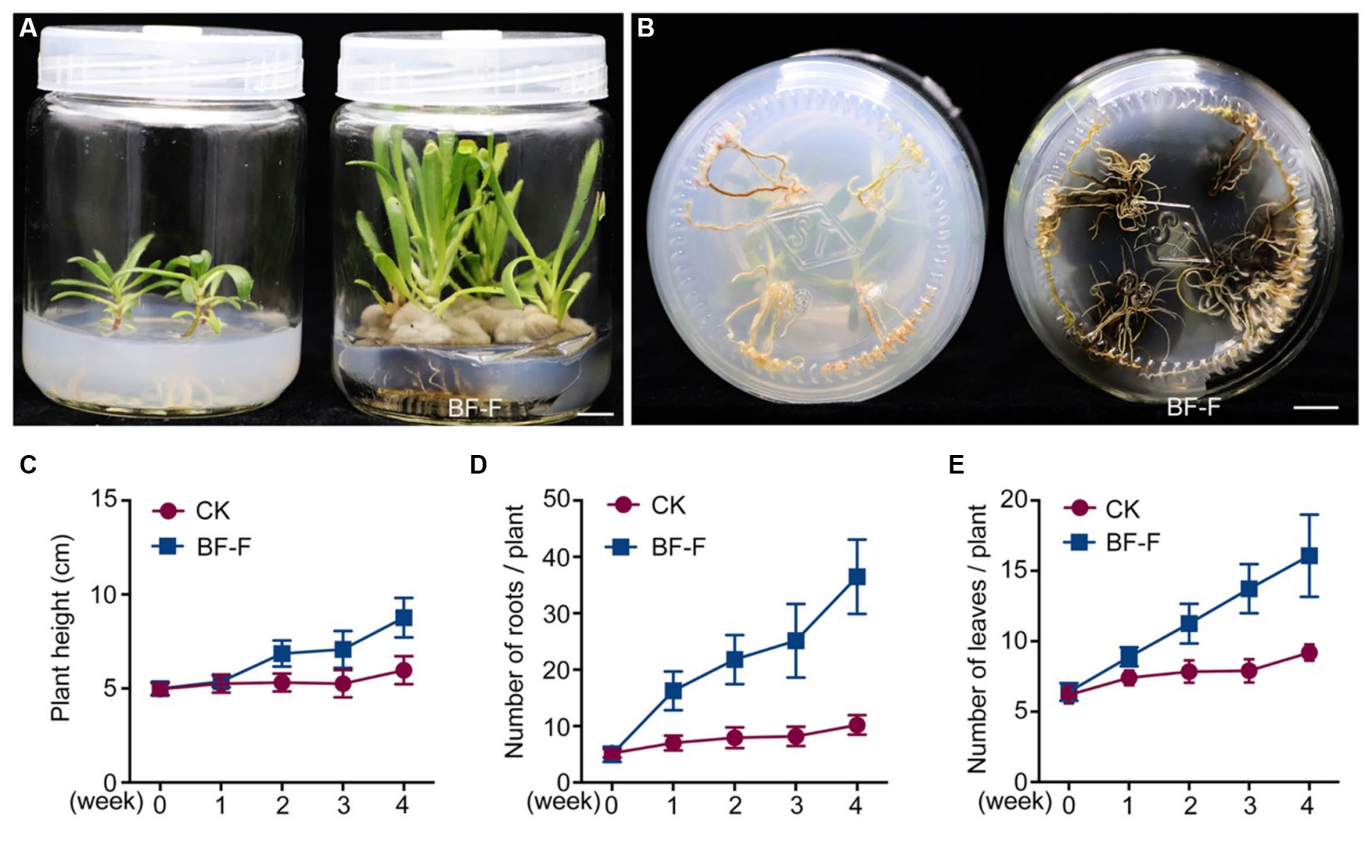
Growth promoting effects of BF-F on *Sesuvium portulacastrum* seedlings. **(A)** Shoot phenotypes of *S. portulacastrum* seedlings after 4 weeks of growth in MS medium uninoculated (left) and inoculated (right) with BF-F. **(B)** Root phenotypes of *S. portulacastrum* seedlings after 4 weeks of growth in MS medium uninoculated (left) and inoculated with BF-F. **(C–E)** Plant height, leaf and root numbers of *S. portulacastrum* seedlings grown in MS medium uninoculated and inoculated with BF-F for 4 weeks. Data represent means ± SD with three biological replicates, each had at least 20 seedlings.

Although some *Cladosporium* sp. are known to promote plant growth (El-Morsy, 2000; Hamayun et al., 2009; Paul and Park, 2013) and enhance plant stress tolerance (Chaibub et al., 2016; Torres et al., 2017), little information is available on *C. angulosum* effects on plant growth. After the inoculation with BF-F, the growth of *S. portulacastrum* seedlings were significantly larger (Figure 3A) and roots were more abundant (Figure 3B) than control. Plant height, root numbers, and leaf numbers were substantially greater than those of plants without BF-F inoculation, and the growth-promoting effects became increasingly different with plant growth time (Figures 3C–E). It was unknown if there was a dose dependent effect on plant growth because BF-F inoculant used in this study was a piece of BF-F fungus colony (0.5 cm). Further study regarding this is warranted.

**Figure 4:**
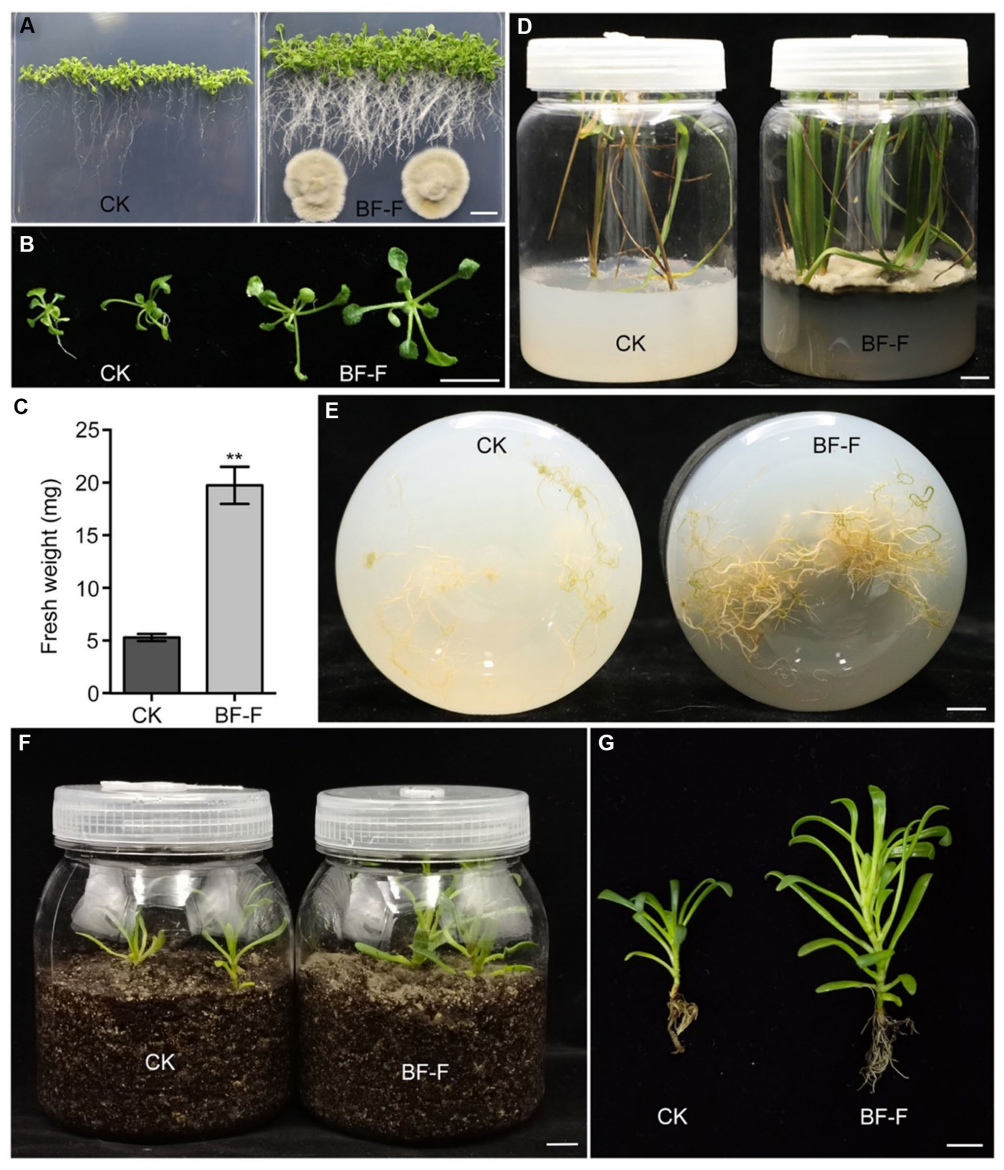
Growth promoting effects of BF-F on different plants. **(A)** Overall growth phenotypes and **(B)** shoot phenotypes of Arabidopsis seedlings grown on MS medium without and with BF-F for 2 weeks. **(C)** Shoot fresh weight of Arabidopsis seedlings after 2 weeks of growth without and with BF-F. Data represent means ± SD of at least 20 seedlings with three biological replicates. **(D)** Growth phenotypes of rice seedlings after uninoculated and inoculated with BF-F for 4 weeks. **(E)** Root phenotypes of rice seedlings after uninoculated and inoculated with BF-F for 4 weeks. **(F–G)** Growth phenotypes of *S. portulacastrum* seedlings growing in soil without and with BF-F for 5 weeks. Bar = 1 cm.

In order to be certain that the growth promotion was not due to specific plant species, BF-F was first inoculated in seedlings of dicot model plant *A. thaliana*. It was found that BF-F also enhanced the growth of *A. thaliana* seedlings (Figure 4A). *A. thaliana* seedling sizes significantly increased (Figure 4B), and the fresh weight of *A. thaliana* seedlings with BF-F inoculation was more than four times greater than that of the control group (Figure 4C). Root elongation appeared to be inhibited by BF-F, but root hairs were extremely abundant with a bushy root system (Figure 4A). Growth promoting effects of BF-F were further evaluated using monocot rice. Results showed that rice seedlings inoculated with BF-F grew more vigorously (Figure 4D) and had more abundant roots than the control (Figure 4E).

### 3.4 General features of the BF-F genome

**Figure 5:**
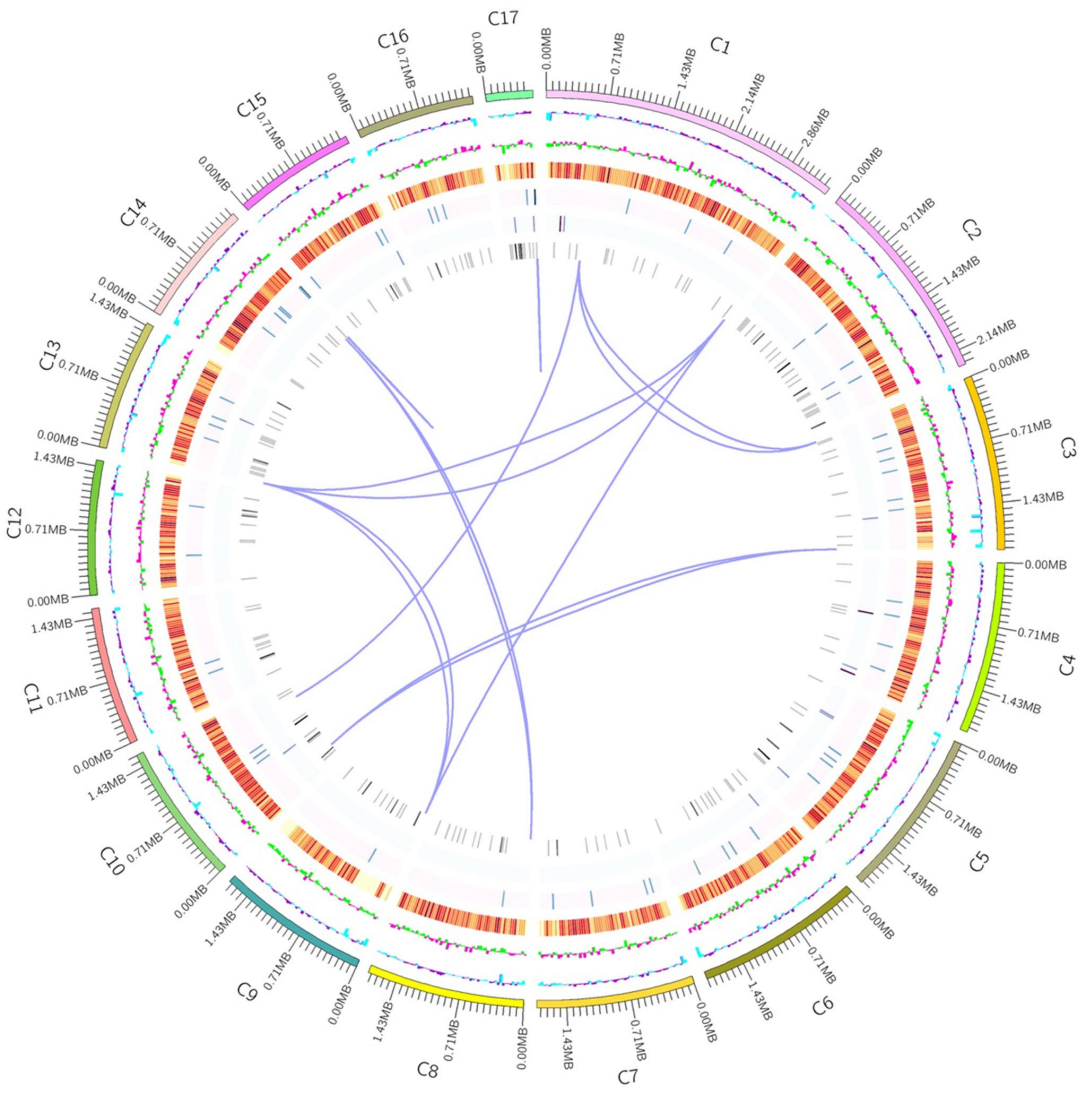
Circular representation of Cladosporium sp. BF-F chromosome. The outer concentric circles show the genome size, gene location, coding number and length of each coding; the middle two circle indicates the % GC content; and the inner circle indicates predicted protein-coding sequences and genes density.

In order to understand the molecular mechanism behind the plant growth promoting effects, the genome of BF-F (GenBank accession number JAMRMS000000000) was sequenced and assembled using Illumina Novaseq 6,000 and PacBio Sequel I. The complete genome of BF-F consisted of a total of 29.4 Mb with a GC content of 52.57%, including 18 contigs (N50 size was 1.75 Mb) (Figure 5). The general genome features are shown in Table 1, of which 6,426 genes were identified as protein-coding genes, with an average length of 1,314 bp. In addition to the predicted genes, a total of 214 tRNA, two sRNA, 18 snoRNA, and 59 rRNA loci (5S, 18S, and 28S) were predicted in the BF-F genome sequence (Table 1).

**Table 1:** General genome features of BF-F.

Feature	Chromosome
Genome size (bp)	29,444,740
Number of Genes	6,425
Gene length (bp)	8,439,339
% GC content	52.57
% of Genome (genes)	28.66
Gene average length (bp)	1,314
% of Genome(internal)	71.34
rRNAs	52,3,4 (5S,18S,28S)
tRNAs	214
sRNA	2
snRNA	18

### 3.5 Gene function annotation

**Figure 6:**
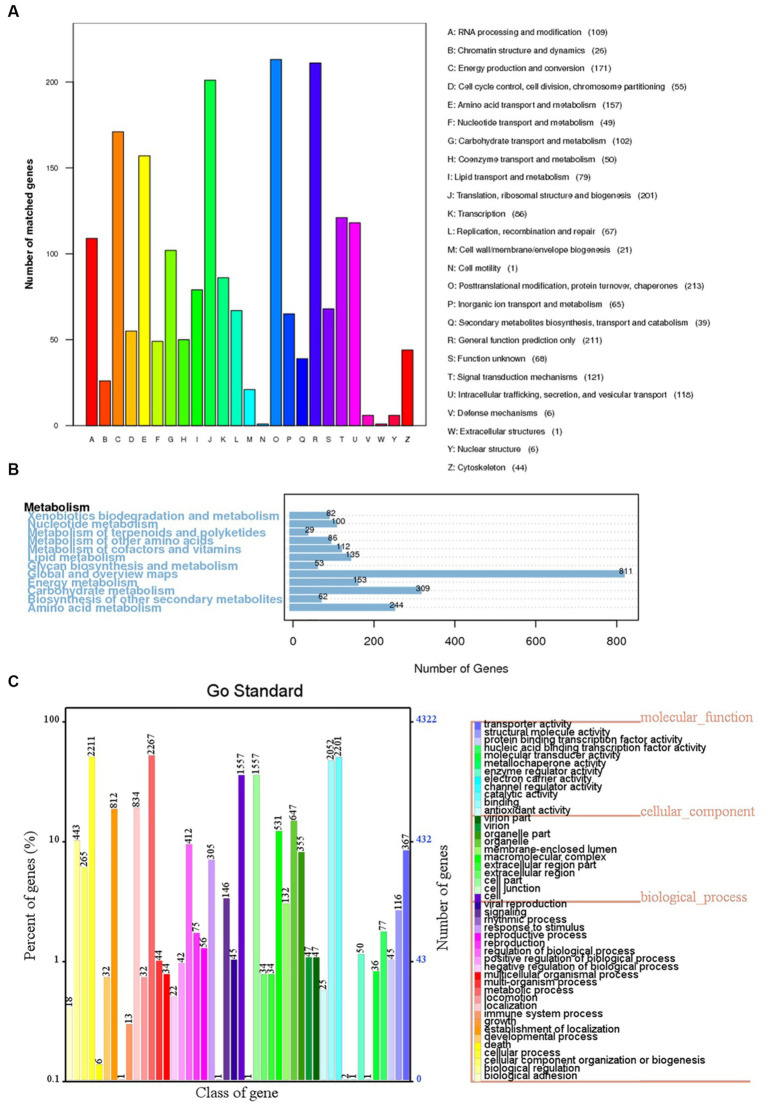
KOG, KEGG and GO classifications of predicted genes in BF-F. **(A)** Distribution of KOG classes for predicted proteins in BF-F. **(B)** KEGG classifications of predicted genes in metabolic pathway. **(C)** Gene Ontology of predicted genes in BF-F. The gene numbers were noted at the right of each bar; The length of each bar represents the percentage of genes in each GO class (the percentage of total GO-annotated genes was set as 100%); Each GO class with number of genes ≤10 was not shown.

The 6,426 predicted genes in the BF-F genome were mapped to the KOG, KEGG, and GO databases (Figure 6). A total of 1,871 predicted proteins were annotated redundantly based on KOG function classification (Figure 6A). Among the highest annotated groups, post-translational modifications of proteins appeared to be significant in cellular regulation, development, and adaptation to stress. Annotation identified 35 putative genes encoding chaperones and 52 genes encoding ubiquitination degradation-related proteins and proteasomes in group O (post-translational modification, protein turnover, and chaperones), which are associated with the regulation of misfolded proteins and metabolic enzymes degraded via the ubiquitin-proteasome system. In group E (amino acid transport and metabolism), tryptophan syntheses and metabolism appeared to be significant. In group G (carbohydrate transport and metabolism), the highest number of genes was related to putative glycolysis/gluconeogenesis.

The KEGG pathway analysis annotated a total of 5,5,572 genes in five metabolic pathways, including amino acid metabolism (348), carbohydrate metabolism (339), overview (335), energy metabolism (195), and metabolism of cofactors and vitamins (195) (Figure 6B). It is not surprising that BF-F contains many genes involved in amino acid metabolism because some amino acids are precursors of plant hormones. For example, IAA is synthesized using tryptophan as the precursor. IAA is a natural auxin and can facilitate the interactions between fungi and other organisms and also promote plant growth (Fu et al., 2015).

According to the GO classification, 4,321 predicted genes received a GO assignment (Figure 6C). A total of 2,267 genes were assigned to the metabolic process category, which may indicate that *Cladosporium* BF-F metabolites are closely associated with plant growth. Therefore, the gene dataset obtained from this study not only enriches the gene database resources but also provides a basis for further investigation of the gene functions of *Cladosporium*.

### 3.6 Genes associated with plant growth-promoting effects

Genome annotation of BF-F revealed several genes contributing directly or indirectly to the promotion of plant growth (Table 2). In the genome annotation, different gene clusters related to IAA biosynthesis were identified. These included tryptophan cluster genes with strong involvement in the biosynthesis (*trpA*, *trpB*, *trpD*, *trpE*, *TRP1*, and *TRP3*) and metabolism (*TDC*, *MAO*, and *ALDH*) of tryptophan. As mentioned above, tryptophan is the precursor of IAA (Table 2). In addition, genes involved in the production of steroids were clustered, and they are implicated in plant growth promotion (Schena et al., 1991; Feldmann, 2006). For example, genes related to plant hormone brassinosteroid (BR) biosynthesis process (Bhar, 2021) were also identified (Table 2). Furthermore, genes involved in nitrogen metabolism and regulation were found in the BF-F genome, including the nitrogen regulation protein cluster (*NRT*, *glnA*, *gtl1*, and *gdhA*) and the nitrite reductase (*NIT-6*) and nitrate reductase (*NR*) clusters (Table 2). Together, these results may imply that phytohormone production and enhanced nutrient absorption in BF-F may contribute to plant growth promoting effects.

**Table 2:** Genes potentially associated with PGP traits in the genome of BF-F.

Function/PGP trait	Gene	Description	Location/locus tag
Tryptophan biosynthesis	*aroQ*	dehydroquinate dehydratase II	Contig10:487946:488395
	*nagZ*	beta-N-acetylhexosaminidase	Contig10:502674:503914
	*QUIB*	qa-3 quinate dehydrogenase	Contig10:1023683:1024675
	*TRP*	tryptophan synthase	Contig10:1441293:1442593
	*TRP1*	prephenate dehydrogenase (NADP+)	Contig10:1496599:1497988
	*trpD*	anthranilate phosphoribosyltransferase	Contig13:1209593:1210753
	*aroF*	deoxy-7-phosphoheptulonate synthase	Contig14:239569:240808
	*aroB*	dehydroquinate synthase	Contig14:540431:542150
	*aroC*	chorismate synthase	Contig16:405385:406659
	*TRP1*	anthranilate synthase / indole-3-glycerol phosphate synthase	Contig1:2203175:2205490
	*trpE*	anthranilate synthase component I	Contig1:2786166:2787913
	*aroF*	3-deoxy-7-phosphoheptulonate synthase	Contig1:321179:322343
	*ARO1*	pentafunctional AROM polypeptide	Contig7:769064:773896
	*GOT1*	aspartate aminotransferase, cytoplasmic	Contig7:1236542:1237861
	*ARO8*	aromatic amino acid aminotransferase I / 2-aminoadipate transaminase	Contig8:394272:396702
Tryptophan metabolism	*DDC, TDC*	aromatic-L-amino-acid/L-tryptophan decarboxylase	Contig3:1013651:1015306
	*ALDH*	aldehyde dehydrogenase	Contig16:1124610:1136277
	*MAO*	monoamine oxidase	Contig1:3334092:3335552
Steroid biosynthesis	*FDFT1*	farnesyl-diphosphate farnesyltransferase	Contig2:34905:36471
	*SQL*	squalene monooxygenase	Contig9:1052550:1054004
	*SMT1*	sterol 24-C-methyltransferase	Contig10:183335:184477
	*CYP51*	sterol 14alpha-demethylase	Contig14:1153188:1154868
	*TM7S*	Delta14-sterol reductase	Contig1:1361362:1362948
	*EBP*	cholestenol Delta-isomerase	Contig5:1697694:1698497
	*SC5DL*	Delta7-sterol 5-desaturase	Contig4:1047053:1048255:-
	*ERG3*	Delta7-sterol 5-desaturase	Contig4:1047053:1048255
Nitrogen metabolism	*NRT*	MFS transporter, NNP family, nitrate/nitrite transporter	Contig10:801362:803187
	*glnA,*	glutamine synthetase	Contig3:216124:217758
	*cyns*	cyanaten lyase	Contig3:5281684:529873
	*GTL1*	glutamate synthase (NADH)	Contig5:744675:751205
	*gdha*	glutamate dehydrogenase	Contig16:221788:223217
Nitrogen reduction	*NR*	nitrate reductase (NAD(P)H)	Contig5:71331:73058
	*NIT-6*	nitrite reductase (NAD(P)H)	Contig8:321642:325025

### 3.7 Hormones and precursors of IAA produced by BF-F

**Figure 7:**
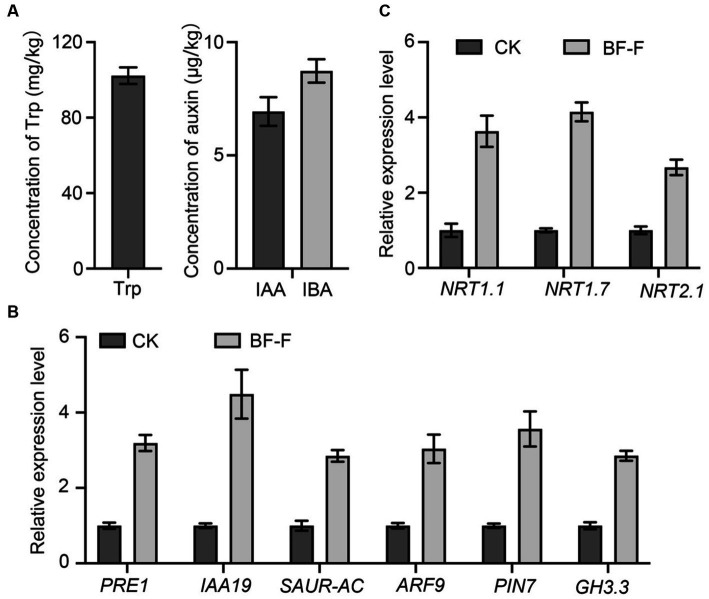
Production of Trp, IAA, and IBA by BF-F and relative expression levels of relevant genes in *Arabidopsis thaliana*. **(A)** Concentrations of Trp, IAA, and IBA in mycelium of BF-F. BF-F was cultured in three flasks containing potato dextrose broth medium on a rotating shaker at 150 rpm, 25°C in total darkness for 5 days. **(B)** The expression level of auxin response and **(C)** N uptake related genes in *A. thaliana* seedlings. *A. thaliana* seedlings grown in MS medium uninoculated and inoculated with BF-F for 2 weeks. *PP2A* was used as an internal control. Data are mean ± SD with three replicates.

In order to determine whether or not BF-F could produce IAA. Mycelium of BF-F was analyzed for precursors involved in IAA biosynthesis. Results showed that Trp was extremely higher with a mean concentration of 100.12 mg kg^−1^ (Figure 7A). Other compounds were detected with IBA at 8.73 μg kg^−1^ and IAA at 6.87 μg kg^−1^ (Figure 7A). qRT-PCR analysis showed that *PRE1, IAA19, SAUR-AC, ARF9, PIN7,* and *GH3.3*, which are responsible for IAA, were highly upregulated in *A. thaliana* seedlings inoculated with BF-F compared to the uninoculated control plants (Figure 7B), and the upregulation corresponded to enhanced growth of seedlings (Figures 4A,B). These results indicated that the production of IAA in BF-F could be an important factor contributing to plant growth promotion.

### 3.8 BF-F promotes N uptake related genes expression in *Arabidopsis* seedlings

Some key genes involved in N uptake, including *NRT1.1*, *NRT1.7,* and *NRT2.7* were analyzed in plants, and results showed that their expression in *A. thaliana* seedlings inoculated with BF-F was significantly higher than those uninoculated with BF-F (Figure 7C). This result indicated that the endophytic BF-F also enhanced plant uptake of N.

## 4 Discussion

This study isolated a new *Cladosporium* fungus strain, referred to as BF-F, from roots of *S. portulacastrum* seedling. It exhibits beneficial effects on plant growth by increasing root and leaf numbers and plant height. Based on the genomic sequence data, BF-F has gene clusters that are implicated in tryptophan biosynthesis and metabolism, which lead to the production IAA and IBA (Figure 7A). It has gene clusters involved in the sterol biosynthesis pathway, which would result in the biosynthesis of phytohormone BR. In addition to phytohormones produced by BF-F that directly contribute to the plant growth promotion, BF-F also support plant growth indirectly by increased expression of genes related to N uptake, such as *NRT1.1*, *NRT1.7*, and *NRT2.1* (Figure 7B). As far as it is known, the genome of *Cladosporium* has not yet been investigated. This study provides the first genomic sequence information about *Cladosporium* species and presents a mechanistic explanation as to how BF-F as a *Cladosporium* strain promotes plant growth. To better understand the growth promoting effects of BF-F, further studies based on the genomic information are warranted.

*Cladosporium* is one of the most common fungal genera distributed in diverse substrates with various lifestyles. The identification of *Cladosporium* species based on morphology has been a challenge. Although conidiophore and conidia size and shape are important characteristics of different *Cladosporium* species, dimensions usually overlap among species in the genus. Therefore, molecular analysis has been used to identify *Cladosporium* species. The ITS rDNA, ACT, and *EF-1α* regions of the genome are often used to explain the diversity and evolutionary trends in the *Cladosporium* genus (Bensh et al., 2010, 2012). However, ITS rDNA alone does not provide acceptable species resolution (Zalar et al., 2007). In contrast, ACT and *EF-1α* demonstrate a high degree of divergence among species (Voigt and Wöstemeyer, 2001; Bensh et al., 2010). In this study an integrated approach based on both molecular and morphological characteristics were used to identify the isolated BF-F. Molecular analysis showed that *EF-1α* sequence of BF-F had only 88% similarity to *C. angulosum* (Figure 2). However, morphologically, BF-F produces no soluble pigment when cultured in PDA medium (Figures 1A,B) and thus differs from *C. angulosum* as it releases sulfur-yellow pigment into PDA (Bensch et al., 2018). On the basis of sequence homology (99%) and phylogenetic analysis, BF-F is likely a new *C. angulosum* strain or possibly a new *Cladosporium* species that is most homologous to *C. angulosum*. Further research is needed to determine its identity.

Endophytic fungi, as an important class of PGPF species have attracted considerable interest due to their multiple beneficial effects on plant growth and improved plant tolerance to abiotic and biotic stresses (Zhang et al., 2006; Meenavalli et al., 2011; Perotto et al., 2012; Murali et al., 2013; Gouda et al., 2016; Toghueo et al., 2017; Strobel, 2018; Wei et al., 2022). Their secondary metabolites also served as an excellent source of bioactive compounds for potential use as antimicrobial, anti-insect, and anticancer materials (Gouda et al., 2016). Plant growth promotion executed by PGPF is a complex process that is often attributed to a variety of direct and indirect factors, including the solubilization of minerals, biosynthesis of growth-stimulating hormones, production of volatile organic compounds (VOCs), increased uptake of nutrients, and improved tolerance to biotic and abiotic stresses (Santoyo et al., 2016; Hossain et al., 2017; Priyadharsini and Muthukumar, 2017; El-Maraghy et al., 2020; Hossain and Sultana, 2020). Many species in the genus *Cladosporium* function as endophytic PGPF through different strategies for plant growth regulation, including gibberellic acid (GA) biosynthesis, the production of volatile substances, or antagonism against plant pathogens (Hamayun et al., 2009; Paul and Park, 2013; Torres et al., 2017). In this study, the isolated BF-F can substantially promote plant growth (Figures 3, 4). The promotion is not only limited to its host plant *S. portulacastrum* but also other dicot plants, such as *Arabidopsis* and monocot rice (Figure 4). Metabolic pathway gene cluster analysis of the BF-F genome showed that the tryptophan synthesis and metabolism pathway happened in BF-F (Figures 5, 7A,B and Table 2), tryptophan metabolite IBA and IAA occurred in BF-F mycelium (Figure 7A), and auxin response genes correspondingly upregulated in *A. thaliana* seedlings inoculated with BF-F. All these results suggest that the production of IAA by BF-F is one of main reasons facilitating plant growth promotion, which has not been reported in *Cladosporium* species thus far. Moreover, sterol synthesis pathways were also found in BF-F genome, suggesting that BF-F has the potential to produce sterols (Figure 5 and Table 2). Although sterols may have limited effects on plants, sterols are the precursors of BR, which promotes plant growth. In addition, the annotated BF-F genome also revealed the presence of several gene clusters involved in nitrogen metabolism and reduction (Table 2). Plant N uptake related genes were upregulated in *A. thaliana* seedlings when inoculated with BF-F (Figure 7C), which may be another mechanism underpinning plant growth promotion executed by BF-F. These new metabolites and metabolic pathways in BF-F may not only explain the molecular mechanisms of plant growth promotion but also probably represent important characteristics for distinguishing BF-F from other *Cladosporium* species.

The investigation of clusters of genes involved in metabolic pathways is an emerging area in plant biology, and research has provided some provocative insights into plant genome plasticity and evolution (Osbourn, 2010; Hautbergue et al., 2018). Clusters of functionally related genes has been investigated in different PGPF and plant growth-promoting rhizobacteria (PGPR). Genes clusters of phytohormone pathways are often obtained in plant growth-promoting microbes. Gene clusters associated with IAA production are most common in PGPF species (Liu et al., 2016; Abdullahi et al., 2021). *Cladosporium* is a diverse family of fungi with various species and functions. Further analyses of this genus based on a large number of isolates collected from different geographical regions are necessary to redefine species borders and growth-promoting potential within *Cladosporium* (Wirsel et al., 2002). However, the genomic information of *Cladosporium* species, especially PGPF, is still lacking. Although some *Cladosporium* species that can produce GA or volatile compounds have been reported, associated gene clusters remain unclear due to the lack of genomic information (Hamayun et al., 2009; Paul and Park, 2013). In the present study, gene clusters associated with IAA production by tryptophan-dependent pathways and sterol synthesis were identified in the BF-F genome (Table 2). These findings indicate that *Cladosporium* also possesses the potential to produce IAA and/or sterols for improving plant growth. Further analysis of *Cladosporium* species genomes will help uncover new metabolites and new metabolic pathways associated with plant growth promotion.

## 5 Conclusion

*Cladosporium* BF-F strain is a broad-spectrum PGPF that can promote growth of both monocot and dicot plants. The genome of BF-F has been sequenced, which should provide the first glimpse into the genetic basis of the plant growth promotion traits of *Cladosporium*. Based on the genomic information, BF-F also has the ability to produce IAA, similar to other PGPF species. The production of IAA along with indirect effects on N uptake may be the basis of BF-F in enhanced plant growth.

## Data availability statement

The datasets presented in this study can be found in online repositories. The names of the repository/repositories and accession number(s) can be found in the article/Supplementary material.

## Author contributions

NY: Data curation, Writing – original draft. WZ: Writing – original draft, Writing – review & editing. DW: Formal analysis, Writing – original draft. DC: Formal analysis, Writing – original draft. YC: Formal analysis, Writing – original draft. WH: Data curation, Writing – original draft. ZL: Data curation, Writing – original draft. XC: Writing – review & editing. GY: Data curation, Formal analysis, Writing – original draft. ZC: Writing – review & editing. JC: Writing – review & editing. XW: Writing – review & editing, Funding acquisition.

## Footnotes

^1^ http://geneontology.org/

^2^ http://www.genome.jp/kegg/

^3^ http://www.ncbi.nlm.nih.gov/COG/
