# Supplementary material for: A novel endophytic fungus strain of Cladosporium: its identification, genomic analysis, and effects on plant growth
Source: Front Microbiol. 2023 Nov 23;14:1287582. doi: 10.3389/fmicb.2023.1287582 (PMC10706132; doi:10.3389/fmicb.2023.1287582)
Supplement: Supplementary file 1 [file Data_Sheet_1.docx]

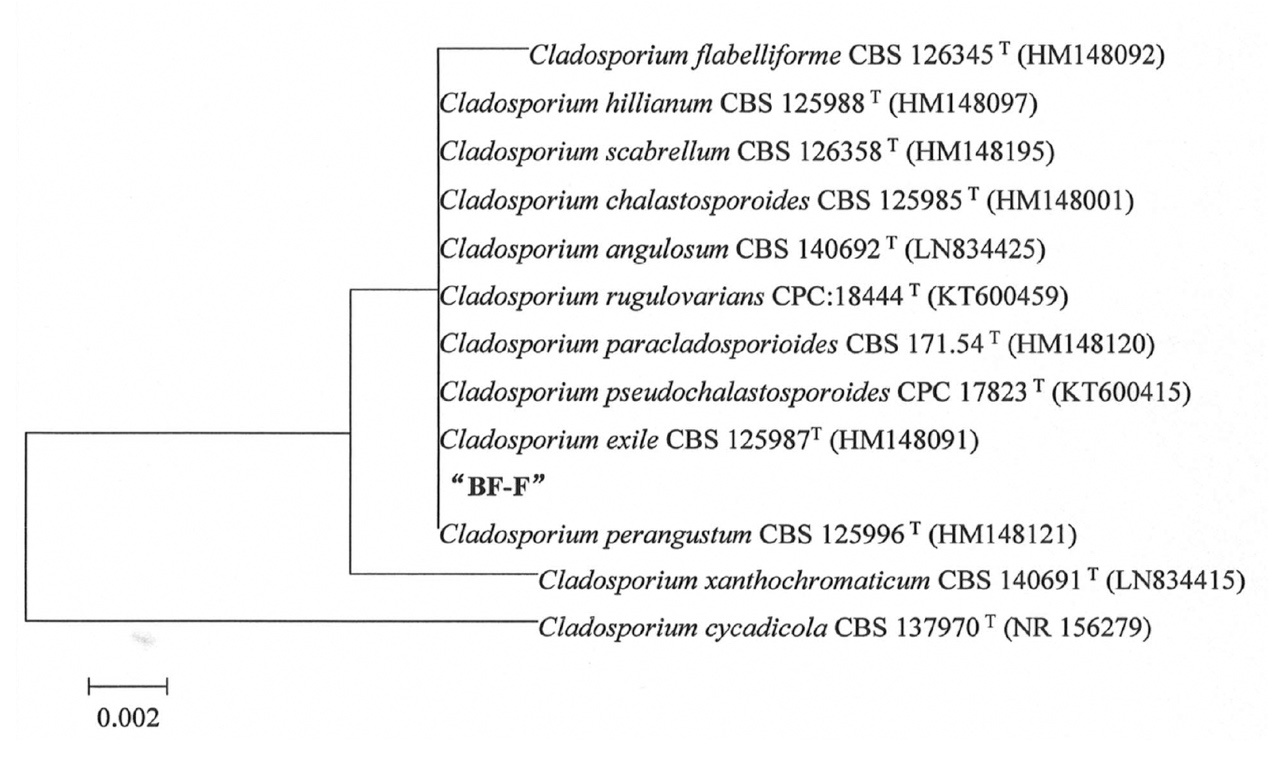


Supplementary Figure 1 Phylogenetic tree analysis of BF-F based on ITS rDNA

Tree topology of BF-F strain based on ITS rDNA sequences using the Neighbour-Joining(N-J) method. The 12 reference sequences were obtained from GenBank. Bootstrap values (calculated from1000 repetitions)≥70% are shown at their relevant nodes and the superscript "T" represented the model strains.

Supplementary Table 1 ITS rDNA and *EF-1α* sequences of BF-F for Phylogenetic tree analysis.

| Region | sequence |
| --- | --- |
| ITS | ATTACAAGTG ACCCCGGTTT ACCACCGGGA TGTTCATAAC CCTTTGTTGT  CCGACTCTGT TGCCTCCGGG GCGACCCTGC CTTCGGGCGG GGGCTCCGGG  TGGACACTTC AAACTCTTGC GTAACTTTGC AGTCTGAGTA AACTTAATTA  ATAAATTAAA ACTTTTAACA ACGGATCTCT TGGTTCTGGC ATCGATGAAG  AACGCAGCGA AATGCGATAA GTAATGTGAA TTGCAGAATT CAGTGAATCA  TCGAATCTTT GAACGCACAT TGCGCCCCCT GGTATTCCGG GGGGCATGCC  TGTTCGAGCG TCATTTCACC ACTCAAGCCT CGCTTGGTAT TGGGCAACGC  GGTCCGCCGC GTGCCTCAAA TCGACCGGCT GGGTCTTCTG TCCCCTAAGC  GTTGTGGAAA CTATTCGCTA AAGGGTGTTC GGGAGGCTAC GCCGTAAAAC  AACCCCATTT CTAAGGTTGA CCTCGGATCA GGTAGGGATA CCCGCTGAAC  TTAAGCATAT CAA |
| *EF-1α* | ATCGAGAAAG TTCGAGAAGG TAAGCCTGAT CATTAGGTCA AGGTGTCCTC  TTTGCAGTGT TCTACCCCTC TGCCCGCCAC GCCACCCCAC CTCGTCGCAA  TCTGCGATAA GATGTGGGAC GCGGCTTGGC TGGGCACGGA CTTCACTGCT  TTGAAGACAG TTACCACACA ACATGACCAT CACAACACAT TACTGACTAC  GACTCCAGGA AGCCGCTGAA CTCGGCAAGG GTTCCTTCAA GTACGCATGG  GTTCTTGACA AGCTCAAGTC CGAGCGTGAG CGTGGTATCA CCATCGATAT  CGCCCTCTGG AAGTTCGAGA CTCCCAAGTA AGTACTCTCA GCGACGCTGC  TATCGCCAGC AAGGAACTTT ACTGCGACGC TTGCGCAAAG CCCTTCAGAG  ATCCGATTGC GCTTAACAGA CACAACAACC AAAGGCATAC ACGCTAACCT  ATTCACCCAC CACAGGTACA ACGTCACCGT CATTGACGCC CCTGGCCACC  GTGACTTCAT CAAGAACATG ATCACTGGTA CCTCCCAGGC TGACTGCGCC  ATTCTCATCA TTGCCGCCGG TACTGGTGAG TTCGAGGCTG GTATCT |

Supplementary Table 2 List of qRT-PCR primers used in this study.

| Gene | Primers for RT-qPCR |
| --- | --- |
| *PRE1-F* | GTTCTGATAAGGCATCAGCCTCG |
| *PRE1-R* | CATGAGTAGGCTTCTAATAACGG |
| *SAUR-AC-F* | AAGAGGATTCATGGCGGTCTATG |
| *SAUR-AC-R* | GTATTGTTAAGCCGCCCATTGG |
| *IAA19-F* | TGGTTCGAGCCAAGGCTATGATG |
| *IAA19-R* | CATCTTTCAAGGCCACACCGATGC |
| *ARF9-F* | CCCCGACCCAGGAGTTAGTA |
| *ARF9-R* | TGTCCCCAGCAACCAATCTC |
| *GH3.3-F* | CATCACAGAGTTCCTCACAAGC |
| *GH3.3-R* | GTCGGTCCATGTCTTCATCA |
| *PIN7-F* | CGGCTGATATTGATAATGGTGTGG |
| *PIN7-R* | GCAATGCAGCTTGAACAATGG |
| *NRT1.1-F* | TAAGGGATCAGGAAGCGGGA |
| *NRT1.1-R* | AAGAGGATGCATGTTGCCCA |
| *NRT2.7-F* | ATGGAGCCATCTCAACGCAA |
| *NRT2.7-R* | AGGTGAAAGGCTCGTGAGTG |
| *NRT1.7-F* | GAAAGCTGAAACTTCCGGCG |
| *NRT1.7-R* | TATAACCACCGCGGCTTTGT |
| *PP2A-F* | TATCGGATGACGATTCTTCGTGCAG |
| *PP2A-R* | GCTTGGTCGACTATCGGAATGAGAG |
